# 1D and 3D co-simulation and self-adaptive position control of electrostatic levitation in China’s Space Station

**DOI:** 10.1038/s41526-022-00215-6

**Published:** 2022-08-02

**Authors:** Peng Zhang, Yang Zhang, Zile Wang, Yang Wang, Mao Li, Ran Niu, Li Liang, Wenju Yang, Ming Gao, Hongen Zhong, Xuzhi Li, Jianding Yu

**Affiliations:** 1 Technology and Engineering Center for Space Utilization, Chinese Academy of Sciences, Beijing, China; 2 State Key Laboratory of High Performance Ceramics and Superfine Microstructure, Shanghai Institute of Ceramics, China Academy of Sciences, Shanghai, China

**Keywords:** Mechanical engineering, Characterization and analytical techniques

## Abstract

The greatest challenge of electrostatic levitation for containerless material processing is the stable control of charged material during heating. Recently, high-precision self-adaptive control of electrostatic levitation has been achieved in China’s Space Station. Based on the 1D and 3D co-simulation analysis, an optimal scheduling of control strategies of sample release and retrieval in space is developed. Both simulation results and on-orbit experiments demonstrated that the inversion of surface charge is responsible for the heating induced material instability. On-orbit experiments indicated that under laser illuminations, the net surface charge of metal Zr changed from positive to negative at 900 K and from negative to positive at 1300 K. The possible physical mechanism of the charge inversion of heated material is discussed.

## Introduction

Containerless material processing using aerodynamic^1,2^, acoustic^3,4^, electromagnetic^5–7^ and electrostatic levitation^8–23^, has been widely used in the study of materials science experiments and thermophysical properties measurements. Containerless methods not only avoid chemical contamination at high temperatures caused by the direct contact with the container, but also eliminate extrinsic heterogeneous nucleation. By so doing, melts can often be supercooled to accesses non-equilibrium states, glasses and otherwise unavailable crystalline forms of materials. Among these levitation methods, electrostatic levitation is more valued due to its broad applicability. Since it levitates the charged object by the electrostatic force, it can accommodate a broad range of materials including metal, alloy, and oxide as long as the sample carries sufficient amount of charge. Additionally, it provides a variety of special environments for conducting material experiment such as high-vacuum, inert gas, and high pressure.

Many thermophysical properties of levitated melts can be measured on Earth, but buoyancy-driven convection often masks underlying diffusion phenomena. The microgravity environment greatly reduces buoyancy-driven convection and provides quiescent conditions for thermophysical property measurements and nucleation. Also, only weak electric field is enough for the stable levitation in microgravity while strong electric field is required to overcome the gravity on the ground. Currently, there are two electrostatic levitation furnaces (ELF) in operation in space, including the ELF developed by Japan Aerospace Exploration Agency, which was launched to the International Space Station (ISS) in 2015, and the China’s ELF launched to Tiangong Space Station in May 2021. Recently, several samples have been stably levitated in the ISS and thermophysical properties of them have already been reported^17^.

The greatest challenge for conducting electrostatic levitation experiments is the accurate position control of test samples using levitation force, especially at the laser-heating stage, where acute sample instability occurs when the material is heated up over a certain temperature. The prior work recognized that responding to changes in the charge on the sample is an essential requirement for maintaining stable levitation during first heat up from ambient and believed that this phenomenon resulted from the surface charge loss of the sample during heating^8–15,22^. Therefore, a system to compensate positive charge for the heated sample is required for keeping levitation stable. A commonly used method to resolve it is to use a high-power deuterium lamp to maintain the positive charge based on the photoelectric effect^9,11–13,16,17^. Photoelectric charging has been used successfully in some ground-based levitators. However, this method is typically not suitable when a gaseous atmosphere is present. The gas can ionize and cause discharges from the high voltage. Alternatively, in the ground-based experiments, the preheating technique can also ensure the control stability but only for materials with melting temperature higher than 1500 K^12,18^.

Various control algorithms were developed to improve system robustness, including fuzzy logic scheme for online tuning of controller parameters and actively identifying the amount of electrical charge in real-time^9,11,15,24,25^. The previous algorithms can actively adjust the controller parameters corresponding to the amount of electrical charge, but they are not valid to deal with the instabilities if the charge polarities flip abruptly. In China’s ELF, instead of using UV lamps to maintain the positive charge, the self-adaptive control strategy has been proposed to resolve the laser heating induced sample instability without having to compensate the surface charge.

As we know, both the time and financial cost can be considerably high in the realization of a new technology, especially for those in space exploration and space science. Therefore, numerical simulation-driven assessments and optimizations is particularly helpful for obtaining an optimal scheduling of control strategies in the complex electrostatic levitation system. In this work, we propose a 1D and 3D co-simulation method and test its validity for optimizing the scheduling of the control strategy on the ELF in China’s Space Station.

## Methods

### Containerless experiment system

**Fig. 1:**
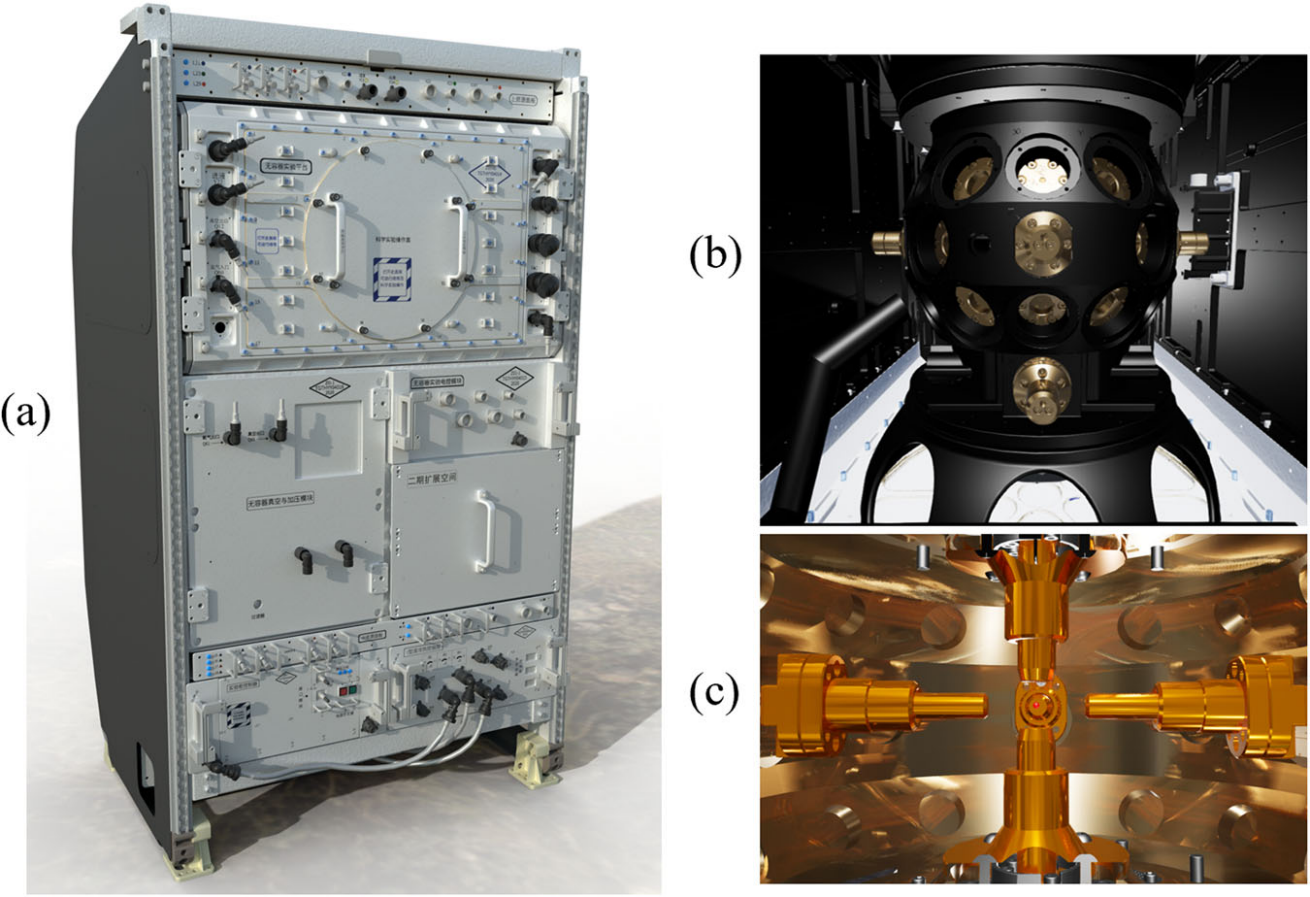
Drawing of electrostatic levitation furnace in China’s Space Station. **a** Containerless material science experiment rack, **b** Experiment chamber with 36 optical windows, **c** Electrode configuration.

The electrostatic levitation furnace in China’s Space Station is shown in Fig. 1. The rack for containerless material science experiments is composed of an experiment chamber and supporting modules for power supply, information recording and cooling. The core module of the rack is the experiment chamber with 36 optical windows. An axial structure including three pairs of cylindrical electrodes that are pairwise orthogonal is nested in the chamber.

**Fig. 2:**
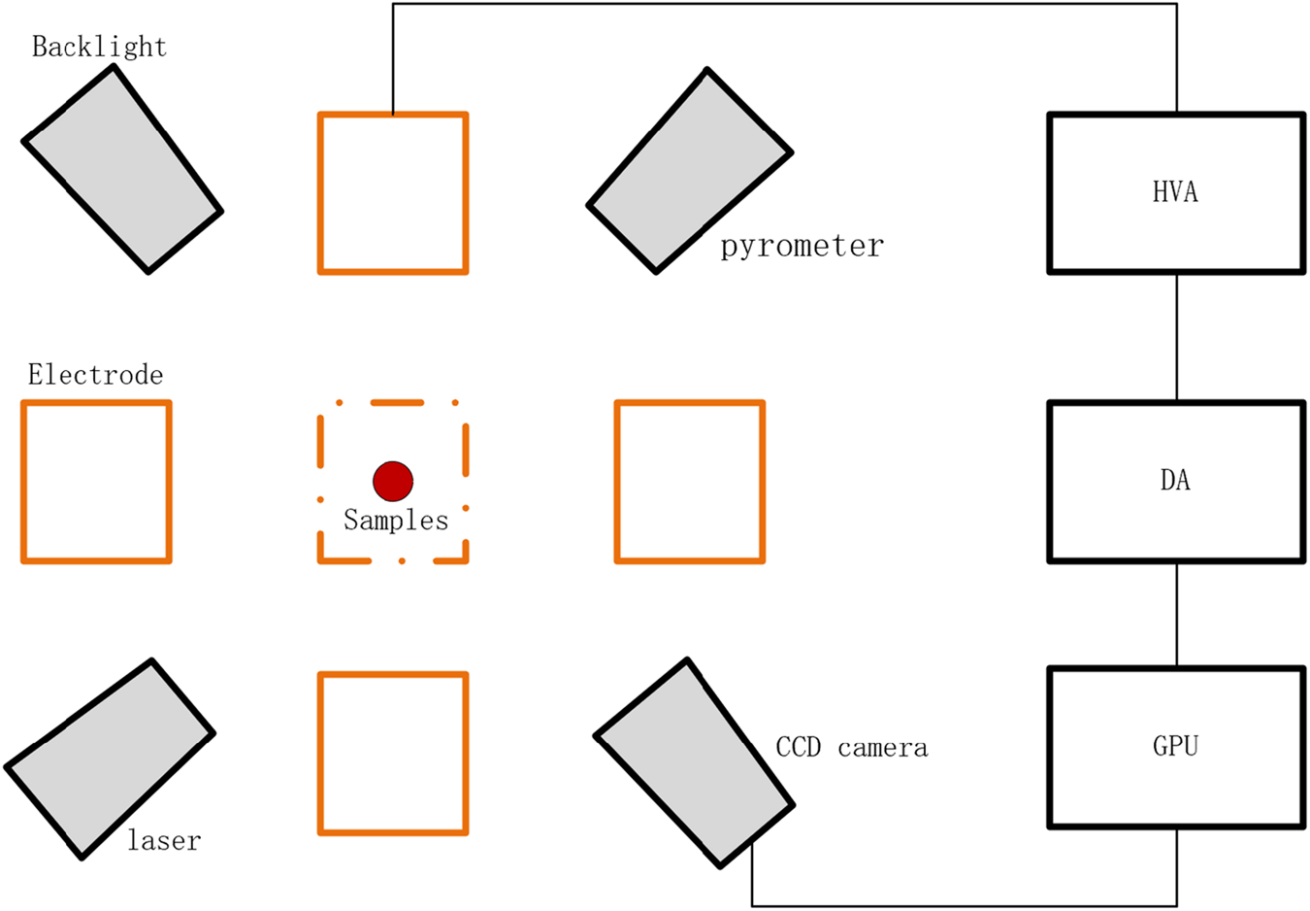
The simple model of electrostatic levitation control system. The charged samples is controlled by the electrostatic force generated by three pairs of high voltage electrodes with a feedback loop.

The stable levitation of charged samples is automatically controlled by the electrostatic force generated by three pairs of high voltage electrodes with a feedback loop. Figure 2 illustrates the simplified model of test sample on *x-z* plane and the control loop of electrostatic levitation system.

Accurate and high-speed position detection is of great importance to electrostatic levitation. PSD (Position Sensing Device) is commonly used in ground-based electrostatic levitators^8^. By contrast, video imaging method has a better signal to noise ratio and can obtain a clearer sample edge by using the algorithm of image identification^16,17^.

In our levitator, the material spatial position is detected by two orthogonal high-speed charge-coupled device (CCD) video cameras (Dalsa M640) in conjunction with two high-intensity backlights (650 nm in wavelength). The high-speed image captured by CCD cameras is analyzed by multi-core graphic processing unit (GPU), and the voltage signal that is required to stabilize material is instantly calculated according to the design of Proportion-Integration-Differentiation (PID) controller. The voltage signal in digital is converted by digital-analog (DA) unit and then the analog signal is fed to high voltage amplifier (Matsusada AS-3B1). Finally, the electric field are generated by three pairs of high voltage electrodes to stabilize charged samples.

The position detection rate is around 700 Hz, due to the limitation of computational ability of GPU for image processing. The change rate of high voltage supplies can reach up to 12 *V* μs^−1^ within the range of ±3000 V. The spatial resolution of the position measurement is around 0.04 mm, which is limited to one pixel change on the camera sensor. The accuracy of levitation position can be controlled within ±0.1 mm with the optimal selection of PID parameters.

The pair of electrodes in the *z* direction are designed to be hollow, which allows two pushing rods transferring samples from the sample cartridge to the levitation area. The diameter of samples should be smaller than 3 mm due to the limitation of the size of sample holder and the hole in the electrode. The two pairs of cylindrical electrodes in the *x* and *y* direction provide the lateral electrostatic forces. The interval between *z*+ and *z*− electrodes is much less than that in the *x* and *y* direction to provide stronger electrostatic force at the stage of sample release and retrieval. The six electrodes are designed in a similar way as described in the ISS-ELF^17^.

Four semiconductor lasers (LD) with the wavelength of 915 nm and one CO_2_ laser with the wavelength of 10.6 μm are used to heat samples. A tetrahedral laser heating configuration for LD lasers was implemented to avoid the destabilizing effect of photon and evaporative anisotropic induced forces^26^. Besides, this multi-beam heating configuration can suppress sample rotation and improve the temperature homogeneity. The maximum optical power of each LD laser and CO_2_ laser is 75 W and 5 W, respectively. All the lasers have a spot size of nearly 1 mm. Metal samples can be heated by only LD lasers, but upon heating oxide materials, CO_2_ laser is also required as generally 915 nm wavelength cannot be absorbed well.

The material temperature is simultaneously measured by a single-wavelength infrared pyrometer (IMPAC IGA 5) and a dual-wavelength infrared pyrometer (IMPAC IGAR 12-LO) with a sampling rate of 500 Hz. The operating wavelength of single wavelength pyrometer is from 1.45 μm to 1.8 μm. The dual wavelength pyrometer is operated at 1.28 μm and 1.65 μm wavelength. Both pyrometers are calibrated to true temperature using the known melting plateau of the material and they have a repeat accuracy of ±1%.

### Experimental procedure

The sealed chamber can provide experimental environment ranging from near vacuum (10^−4^ Pa) to highly pressurized environment (300 kPa) with inert gas (Ar) for the processing of metal and oxide materials. Although a purity (99.999 at. %) Ar atmosphere can be used to study Zr, possible chemical reactions between the highly reactive molten metals and residual gas could alter the intrinsic surface tension or viscosity values. Very small amounts of oxygen can decrease the surface tension of metals^27,28^. Hence, we conduct the experiments of Zr samples under a ~10^−4^ Pa vacuum condition.

The technique for sample release and retrieval in microgravity experiments is first proposed in the ISS-ELF^16,17^, where a rod is used to push samples to hit the bottom electrode for initial charging and the sample is retrieved by using electrostatic force. However, the hitting approach has some uncertainty in the position and velocity after the bounce of charged sample, which may cause the loss of control. Accordingly, we design a new approach for the levitation initiation.

The Zr samples are ~2.7 mm in diameter and are firstly transferred by the pushing rods from the sample holder to the hole of the *z*+ electrode. A constant high voltage signal is then fed to the *z*+ electrode. Under electrostatic attraction, the sample contacts the bottom electrode and gets like electric charges by contact. After a few seconds, the sample is electrostatically repelled from the *z*+ electrode. Once the charged sample enters the control area, its position is detected by the CCD camera and the feedback control starts to work.

When the sample is controlled in the right position, four LD lasers is used to heat samples and a self-adaptive control algorithm is enabled to actively identify the charge polarity of heated samples. Following the thermophysical property measurements of the melt, the heating lasers are turned off and the melts solidifies rapidly.

Finally, the processed samples are retrieved to the sample holder by constantly supplying the bottom electrode with a high voltage, so it repels the charged sample quickly into the hole of the top electrode.

It is worth noting that no UV lamps are used to maintain the positive surface charge during heating. Instead, a well-designed self-adaptive control strategy is utilized to avoid the laser heating induced material instability. The above optimal scheduling of experimental procedure and control strategies is based on a series of simulation-based analyses and is proven to be extraordinarily effective in on-orbit experiments.

### 1D and 3D co-simulation method

To obtain optimal control strategy and a robust control system, high-precision numerical simulation of electrostatic levitation is necessary. System dynamics simulation tools such as Simulink are usually used for modeling one dimensional (1D) complex control systems^11,19^ due to the short computing time. Considering the lack of accuracy in 1D system model when estimating the electrostatic force between charged samples and high voltage electrodes^20,21^, performing a three dimensional (3D) electrostatic simulation to obtain an accurate electrical force could significantly improve the overall accuracy of 1D system model. Therefore, a coupled 1D and 3D co-simulation approach is very attractive considering their complementary advantages and is to be implemented in the present work.

**Fig. 3:**
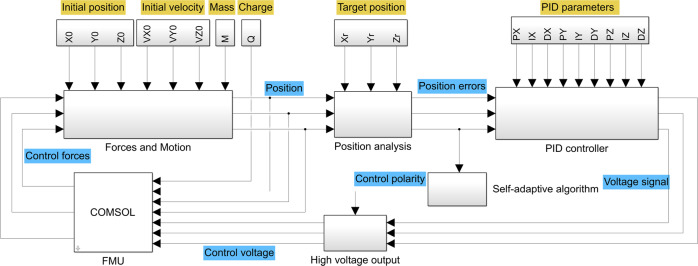
1D and 3D co-simulation model. It implements system model in Simulink and 3D electrostatics model in Comsol Multiphysics.

The co-simulation proposed here is the implementation of 1D control system modeling in Simulink and the 3D electrostatic modeling in COMSOL Multiphysics version 5.6, as illustrated in Fig. 3. The method uses the Simulink system model as the simulation master, while the 3D model is used as a sub-model with more detailed physical information of electric field and electrostatic force, which is represented by a Functional Mock-up Unit (FMU). COMSOL is integrated into Simulink via the LiveLink for Simulink, which can co-simulate using FMU files in a Simulink diagram. As shown in Fig. 3, the Simulink inputs the position and the voltage information of the electrodes to the COMSOL model while the COMSOL model subsequently computes the electrostatic force applied on charged samples and feeds it back to the Simulink.

According to the detected position of test samples, the voltage of the electrodes is computed by the PID feedback with

1 $$U_i\left( k \right) = K_{P_i}e_i\left( k \right) + K_{Ii} {\sum e_i\left( k \right)} + K_{Di}\left( {e_i\left( k \right) - e_i\left( {k - 1} \right)} \right),i = x,y,z$$

where $e_i\left( k \right) = x_i^r - x_i\left( k \right)$ denotes the position deviation in the $k^{th}$ loop. $x_i^r$ is the target position and *x*_*i*_ is the center position of the levitated sample. $K_{P_i}$, *K*_*Ii*_ and *K*_*Di*_ are the PID parameters.

The motion of the levitated object is described by

2 $$m\ddot x_i = mg_i + F_i$$

where *m* and $\ddot x_i$ are the mass and the acceleration, respectively, *F*_*i*_ is the electrostatic force and *g*_*i*_ is the gravitational acceleration in space.

In the Simulink system model, the error and time delay in position measurement as well as ripple voltage are taken into consideration to improve the accuracy of simulations. The COMSOL package enables an accurate computation of the electrostatic force of a charged sample. Consider a charged conductive sphere in the electric field of six cylindrical electrodes. The electric potential *ϕ* is described by the Poisson equation

3 $$\nabla ^2\phi = 0$$

The boundary conditions of the six electrodes are given by setting the potential as

4 $$\phi = U_i^ \mp$$

where $U_i^ \mp$is computed in the Simulink system model. The initial surface charge of the conductive sphere is given according to the electrostatic simulation.

### Reporting summary

Further information on research design is available in the Nature Research Reporting Summary linked to this article.

## Results

### Electrostatic force calculation

A charged sample placed in an electric field is subjected to two competing electrostatic forces, Coulomb force and electrostatic adhesion force. The Coulomb force equals the product of the amount of net charge on the sample and the electric field strength. The electrostatic adhesion force results from the image charges on the electrodes produced by the charged sphere. Assuming a uniformly distributed electric field and a point charge, the electrostatic force of a sample with charge *Q* in the *z* direction can be calculated by the analytical expression,

5 $$F_z = F_{Coulomb} + F_{adhesion} = Q\frac{{U_z}}{{d_z}} + \frac{{Q^2}}{{16\pi \varepsilon }}\left[ {\frac{1}{{\left( {d_z/2 - z} \right)^2}} - \frac{1}{{\left( {d_z/2 + z} \right)^2}}} \right]$$

where *U*_*z*_ is the potential difference between *z*+ and *z*− electrodes, *d*_*z*_ is the distance between the two electrodes, *z* is the center position of samples. When the charged sample is located at the center, the adhesion force vanishes, as indicated in Eq. (5).

**Fig. 4:**
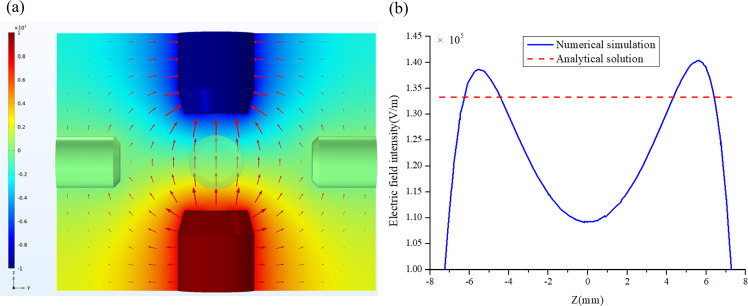
Numerical simulation of electric field. **a** Non-uniformly distributed electric field; **b** Electric field intensity distribution along *z* axis compared with the constant value computed by $E_z = U_z/d_z$.

In fact, the radius of test samples is around 2–3 mm, which is comparable to the radius of electrodes. Resultantly, the electric field is completely non-uniform and the electrostatic force estimated by Eq. (5) is inaccurate. Here we give an example of numerical simulation to illustrate the non-uniform distribution of 3D electric field. Consider the case that the electric potential of *z*+ and *z*− electrodes is −1000 V and 1000 V, respectively, and that of the other four electrodes is zero. The simulation results are shown in Fig. 4a, where the colors represent the electric potential and the arrows indicate the electric field direction in the *y-z* plane. The electric field on the *z* axis has only the *z* component and the intensity distribution *E*_*z*_ along *z* axis is plotted in Fig. 4b. *E*_*z*_ changes nonlinearly with *z* coordinate. The maximum value appears at two sides close to *z*+ and *z*- electrodes and it has the minimum value at the center. The constant value computed by $E_z = U_z/d_z$ is also plotted in Fig. 4b (solid blue line) as a comparison. Obviously, this simplified expression cannot accurately capture the electric field behavior and thus the 1D Simulink model co-simulated with 3D electrostatic model is essential for high-precision simulation of an electrostatic levitation system.

**Fig. 5:**
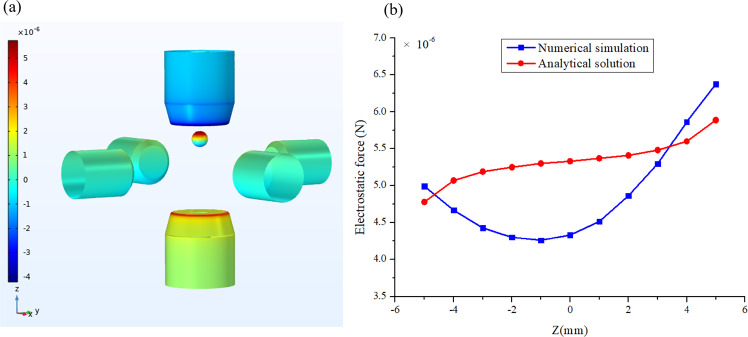
Simulation results of surface charge and electrostatic force. **a** Surface charge density distribution and **b** Electrostatic force component *F*_*z*_ of conductive sphere with net surface charge *Q* = 4e−11 C, in comparison with the analytical results of Eq. (5).

Figure 5a shows the 3D simulation results of surface charge density distribution of a conductive sphere with net surface charge *Q* = 4e−11 C. The electric potential of three pairs of electrodes is the same as that in Fig. 4. Figure 5b plots the simulation results of the *z* component of electrostatic force for charged samples at the different position of *z* axis, which is compared with analytical results computed by Eq. (5). Obviously, there exists a large gap between the 3D simulations and the simplified model, especially at the center position. Hence, the 3D electrostatic simulation to compute the electrostatic force is necessary in the co-simulation frame to improve the overall control model.

### Co-simulation results for sample release and retrieval

**Fig. 6:**
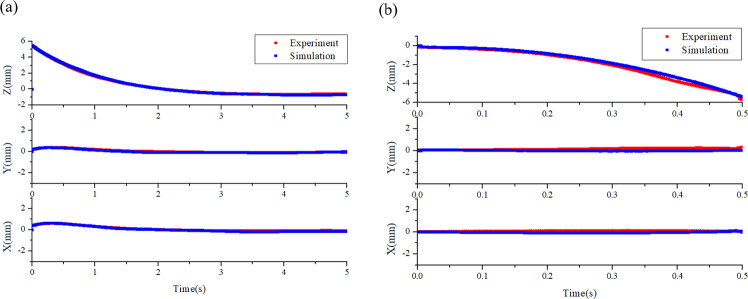
Simulation results of position change of charged samples. **a** Sample release and **b** sample retrieval. Simulation results and on-orbit experiments of sample position show a good agreement. *Q* = 4e−11C.

In the previous works, the amount of the surface charge on the sample was estimated by fitting the sample position profile with simulation results. However, the net surface charge may be underestimated since the actual electrostatic force is much less than that predicted by the analytical solution where the electrical field is assumed linearly distributed, as illustrated in Figs. 4 and 5. Here, the initial amount of surface charge on conductive metal Zr is calculated by 3D finite element calculation and then is verified by the comparison of co-simulation results with the sample position profile, as shown in Fig. 6.

The samples initially get the surface charge by contact charging. Once it contacts with the bottom electrode, they become equipotential in the electrostatic state. In the on-orbit experiments, the initial electric potential of the bottom electrode is set as 1000 V or −1000 V and the others are zero. It can be calculated from the 3D electrostatic simulations that the net surface charge *Q* is ±4e−11C.

Using the 1D and 3D co-simulation method, the sample position profile during sample release and retrieval is numerically simulated. The initial position and velocity of charged samples are determined by the high-speed CCD camera. The comparison shows good agreement between the on-orbit experimental results and the co-simulation results, as plotted in Fig. 6.

The co-simulation model is also utilized to estimate control stability for samples with different amount of surface charge. The levitation stability can be controlled within ±0.1 mm with the optimal selection of PID parameters. The position shaking will increase with the decrease of surface charge *Q*. The simulation results reveal that if *Q* is less than 1.5e−12C, the shaking of sample position will exceed 1 mm, and that if *Q* is less than 1e−12C, the amplitude of sample shaking will gradually increase until the sample loses its control in tens of seconds. Obviously, the decrease of net surface charge will not lead to abrupt instabilities in microgravity environment.

Besides, the microgravity environment greatly reduces the convection and provides a nearly diffusion-controlled transport condition for thermophysical property measurements and nucleation. Excessive sloshing of liquid samples could disturb the quiescent condition and negate some important benefits of low gravity operation. The simulation results show that the acceleration level induced by the control forces is less than 0.1 m s^−2^ when the sample maintains stable levitation.

### Heating induced charge inversion

Although the stable control of charged samples during sample release and retrieval has been achieved based on the co-simulation analysis, a material instability occurred in the on-orbit experiments once the sample of metal Zr is heated up to a certain temperature. The material instability during heating has been widely reported before, the intrinsic nature of charge change for heated samples remains unclear.

Previously, the control instability of heated material was ascribed to the loss of positive surface charge by the possible evaporation of surface contaminants and anisotropic optical pressure^12,13,17,18^. We have estimated the anisotropic optical pressure of uneven laser beams. The optical force acted on the samples are less than the electrostatic forces with one order of magnitude. The co-simulation results also confirmed that the anisotropic optical pressure and the decrease of positive surface charge will not lead to the position instabilities in a zero gravity environment. Obviously, the heating induced position instabilities observed in our experiments are the result of a charge inversion, but not simply the loss of the initial surface charge.

A commonly used method to resolve material instabilities is to use a high-power UV lamp to maintain the positive charge based on the photoelectric effect. In consideration of safety and energy cost, a self-adaptive control to accommodate the charge inversion is developed in China’s ELF instead.

The charge inversion accompanied with position instabilities have been observed in two different experiments. If the sample of metal Zr is positively charged before heating, the charge inversion can be observed at two critical temperatures. The first critical temperature is around 900 K, at which Zr changes its net surface charge from positive to negative. Upon heating to around 1300 K, the net surface charge changes back to positive. At higher temperature, Zr remains positive surface charge. If initially Zr contacts the electrode with negative high voltage and obtains a negative surface charge, the charge polarity is flipped from negative to positive at around 1300 K. The surface charge can be rather unstable from 1300 K to 1500 K sometimes. Over 1500 K, metal Zr always maintains the positive surface charge even for melting.

The charge polarity always remains unchanged at the cooling stage. The change of charge polarity is inevitable at the critical temperature once the sample has never been heated to the critical points. For example, if Zr is cooled from a temperature higher than 1500 K, the surface charge always remains positive when heated again, while if Zr is cooled from a temperature lower than 900 K or 1300 K, the surface charge will flip at 900 K and/or 1300 K.

### Self-adaptive control strategy

To accommodate the charge inversion during heating, a self-adaptive control strategy is developed. The basic principle is not complicated. At the stable levitation stage, the material position fluctuation can be controlled within ±0.1 mm. When the net surface charge inverses, the material will deviate from the target position. Thus, a position threshold larger than 0.1 mm can be used as the judging condition of the charge inversion. In our system, if the deviation exceeds 0.3 mm, the surface charge is considered inverted. Accordingly, the inverse electric field is applied to restabilize the material. This simple strategy has been demonstrated very successful for the stable control of Zr at 900 K, but it is not good enough for the stable control at 1300~1500 K, where multiple charge inversion may occur. The appearance of multiple charge inversion is due to the drop of temperature when the sample position exceeds the laser illumination region. Accordingly, a more complicated control strategy is developed to improve the robustness of this control system. Except for the position threshold, a velocity threshold of 8 mm/s is also designed to restabilize the samples for the case of multiple charge inversion. Besides, a frequency division is utilized to avoid the noise of high-speed detection of position and velocity information. The validity of the self-adaptive control algorithm has been examined by using the co-simulation model.

### On-orbit heating experimental results

**Fig. 7:**
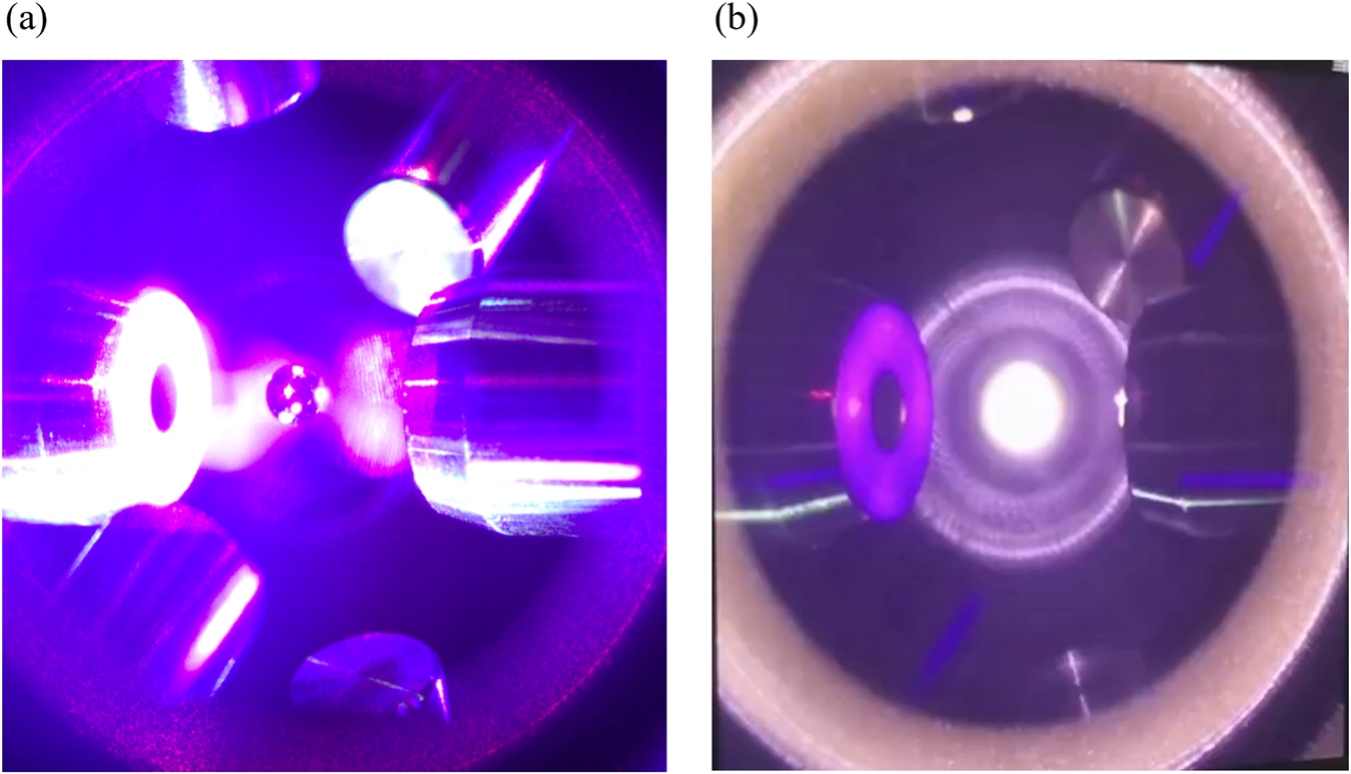
Snapshots from on-orbit experiments. **a** Zr sample before heating and **b** after melting.

**Fig. 8:**
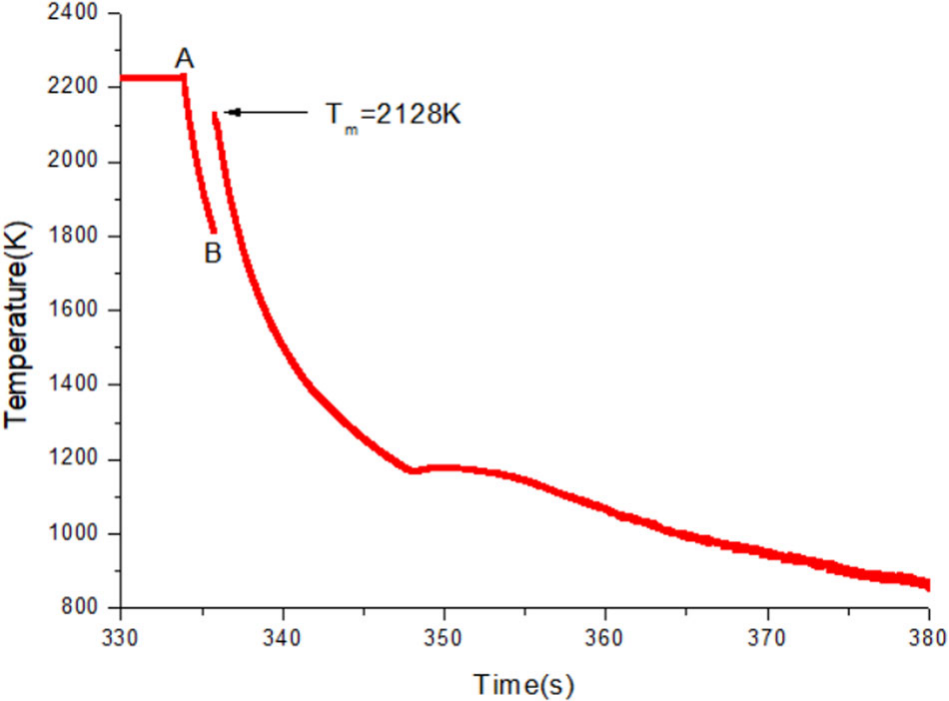
Temperature profile of molten Zr sample. It shows supercooling and recalescence. Heating lasers were turned off at point A and recalescence occurred at point B.

After using the self-adaptive control algorithm in microgravity experiments, the material instabilities caused by the charge inversion are successfully resolved. The snapshots of Zr sample before heating and after melting are given in Fig. 7. Figure 8 depicts a typical temperature profile for a molten pure Zr sample after the laser beams were turned off. The temperature was measured by the single-wavelength infrared pyrometer and it was calibrated to true temperature using the known melting point of pure Zr. Without heating source, the sample was cooled by radiation. A 300 K undercooling and recalescence is observed, which is marked by a sudden temperature rise due to the release of the latent heat upon solidification.

**Fig. 9:**
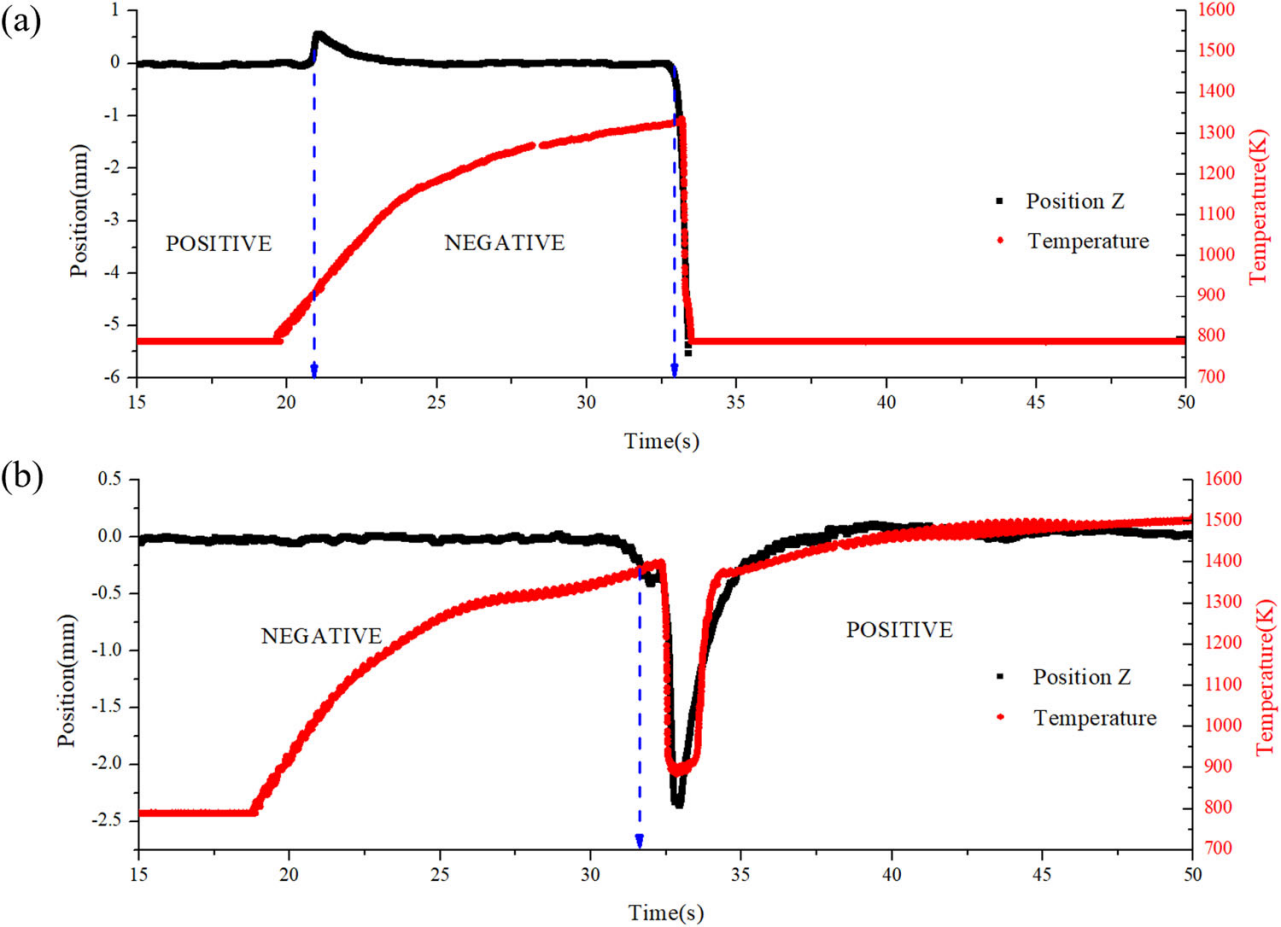
Self-adaptive control during heating. **a** Metal Zr is initially charged with positive surface charge. **b** Metal Zr is initially charged with negative surface charge. The arrows indicate the positions where the control polarities were flipped.

Figure 9a shows the self-adaptive control of an initially positively charged sample during heating. Before heating, the samples are stable at the zero position. After the temperature reaches 900 K, a small position shaking within 0.6 mm occurs, which indicates that the surface charge changes from positive to negative. Once the position deviation exceeds the threshold, the material with negative surface charge is pulled back to the zero position. However, at 1300 K, we lose the control of the sample due to its complicated charge change. Nevertheless, it has been demonstrated by a large number of experiments that the control instability occurred at 1300~1500 K can be very successfully dealt with by utilizing the proposed self-adaptive control strategy if the initial sample is negatively charged.

As shown in Fig. 9b, a large position shaking up to 2.3 mm can be observed when it is heated up to 1400 K, indicating that the surface charge changes from negative to positive. The sharp reduction of temperature is because the sample deviates from the laser illumination region. Obviously, this behavior is quite different from that at 900 K in Fig. 9a. The large position deviation and the drop of temperature increase the difficulty in the position control at 1300~1500 K. After the recovery of stable levitation by the self-adaptive control, the temperature rises up again.

## Discussion

Although the material instabilities during heating in electrostatic levitation experiments has been resolved by different techniques, the intrinsic physical mechanism of the change of net surface charge of metal Zr has not been fully understood. As we know, the sample obtains the initial surface charge by contact charging and after 1500 K the positive net surface charge is maintained due to the sufficient hot electron emission. However, we know little about the negative net surface charge of heated metal Zr from 900 K to 1300 K according to the relevant literatures.

The photoelectric effect depends on the effective work function for the surface, which is the energy barrier for electron ejection. The estimated work function of pure Zr is 4.05 eV^29^. The wavelength of backlights and LD lasers in our levitator is 650 nm and 915 nm. These wavelengths produce photons of energy 1.9 eV and 1.35 eV, respectively. Obviously, they are not energetic enough to ionize the surface of Zr samples in our experiments. Besides, the photoelectric charging is very time consuming and only increases the positive surface charge. Therefore, the change of surface charge caused by photoelectric charging is neglected.

**Fig. 10:**
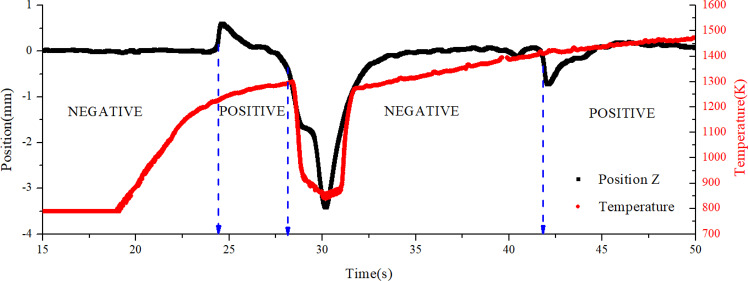
Multiple charge inversion between 1200 K and 1500 K. Metal Zr is initially charged with negative surface charge. The arrows indicate the positions where the control polarities were flipped.

Here we suggest a possible mechanism for generating negative net surface charge and leave the confirmation in the future work. Although the samples are heated in a high-vacuum environment, the residual gas molecules will be ionized to produce negative oxygen ion in the electric field of high voltage electrodes. The negative oxygen ion can be absorbed on the outer layer of the oxidation film and diffuses into the inner layer of the oxidation film. Some defects, such as oxygen vacancies, are formed on the surface during the oxidation process of metal Zr. When it is heated to around 900 K, zirconium dioxide (ZrO_2_) partly dissociates to produce free oxygen diffusing through oxygen vacancy defects. That means ZrO_2_ becomes a solid electrolyte for oxygen. As even more negative oxygen ions enter the material, the amount of absorbed negative charge could exceeds the initial positive charge, which gives rise to the charge inversion at 900 K. When the temperature reaches 1300 K, the emission of hot electron begins to dominate and the net charge becomes positive again. However, the emission of hot electron is highly sensitive to the change of temperature. Once the sample is deviated from laser heating region, the temperature drops quickly. As a results, the hot electron emission becomes weaker and the material may recover the negative surface charge. This behavior heightens the difficulty in the control using self-adaptive control. Figure 10 shows an example of heated metal Zr with multiple charge change after 1300 K. The sample is initially charged with negative surface charge. When it is heated to 1300 K, two small position shaking and a large deviation accompanied with a drop of temperature can be observed, indicating the complex charge change from 1300 K to 1500 K.

High-precision self-adaptive control of electrostatic levitation for containerless material processing has been achieved in China’s Space Station. The control strategy for stable levitation during sample release and retrieval is optimized based on the proposed 1D and 3D co-simulation. Self-adaptive control strategy is utilized to resolve the material instabilities during heating. Moreover, we have shown for the first time that the control instability of heated samples is in fact the result of the inversion of net surface charge, rather than just the loss of initial positive surface charge by the evaporation of surface contaminants or the anisotropic optical pressure.

The positive or negative surface charge of materials at the initial heating stage depends on the initial contact potential before sample launch. Upon heating, the initial positively charged metal Zr becomes negatively charged at the critical temperature of 900 K. If temperatures continue to rise, the surface charge changes from negative to positive at 1300 K. Over 1500 K, metal Zr maintains positive surface charge since the hot electron emission is sufficient, even for the melting and solidification process. If metal Zr is negatively charged before heating, the net surface charge changes from negative to positive only at 1300 K. No charge inversion occurs at the cooling stage for any temperature. If metal Zr is cooled from a temperature higher than 1500 K, the surface charge maintains positive upon heating. However, if it is cooled from a temperature lower than 900 K or 1300 K, the charge inversion is inevitable when heated again.

A possible explanation for the inversion of net surface charge from positive to negative for metal Zr at 900 K is the adsorption of negative oxygen ion in the environment. The inversion from negative to positive at 1300 K is due to the hot electron emission. However, the hot electron emission is very sensitive to the temperature and a large deviation will cause the drop of temperature. As a result, the competition of the adsorption of negative oxygen ion and the hot electron emission can give rise to the multiple charge inversion between 1300 K and 1500 K.

## Supplementary information

Reporting Summary Checklist

## Data Availability

All data presented in the plots are available from the authors upon reasonable request.

## Code availability

The code used to generate the plots is available from the authors upon reasonable request.
